# Expression and the Unconscious

**DOI:** 10.3389/fpsyg.2017.02162

**Published:** 2017-12-12

**Authors:** Jasper Feyaerts, Stijn Vanheule

**Affiliations:** Department of Psychoanalysis and Clinical Consulting, University of Ghent, Ghent, Belgium

**Keywords:** first-person authority, expression, unconscious, Wittgenstein, Freud, Lacan

## Abstract

In the present essay, we aim to develop an expressivist reading of the phenomenon of first-person authority and the adverbial meaning of unconsciousness. In the first part, Wittgenstein's grammatical remarks on the asymmetry between the first -and third-persons in psychological self-ascriptions are developed as an alternative to detectivist explanations according to which first-person authority is to be regarded as a matter of epistemic accomplishment. In the second part, this expressivist account will be used to propose a non-epistemic analysis of the meaning of unconsciousness and to offer a critical discussion of both Freud's and Lacan's respective readings of the unconscious. Regarding the latter, we will reject the idea that the concept of the unconscious (i) necessitates the introduction of a (Cartesian) “subject of the unconscious” and (ii) could be deduced from the paradoxes of first-personal reference.

## Introduction

A recurrent effort that has spurred much discussion in post-Cartesian^1^ philosophy of mind has been to defend Descartes' basic insights on first-person authority while avoiding the tenacious image of what Dennett derisively called the “Cartesian Theater” (Dennett, 1991). That is, most philosophers would agree that I am often able to say what is going on “in” my mind, what I want, believe, desire, intend and so on; whether I like to watch television, feel pain or find your joke funny, without defending the claim that I am able to do so by some sort of mysterious process of “inner perception” or “inner sense” involving ghostly spectators and shadowy images. A second and closely related claim is that this presumed first-person authority is in some important sense different from our third-person entitlements when speaking on behalf of others, or, similarly, when speaking in such a third-personal mode on behalf of ourselves. While it is not immediately clear whether this difference is to be cashed out in epistemic terms—i.e., the claim that self-knowledge always surpasses the knowledge I might have of the other's mental happenings—at least it seems to be a common assumption that there is a basic *first/third-person asymmetry* in the very way I ascribe such happenings to myself or to others which ought to be respected. Indeed, the success and validity of philosophical theories dealing with the perennial issues of subjectivity and first-person avowals is often measured in precisely those terms: they should (i) be able to preserve and explain the first/third-person distinction without (ii) succumbing to the Cartesian picture.

Now there seems to be a broad family of philosophical positions that try to accommodate these requirements by turning to the concept of *expression*. Thus, for example, Wittgenstein's notorious “plan for the treatment of psychological concepts” (Wittgenstein, 1981, §472) invites us to consider first-personal avowals such as “I'm expecting an explosion” or “I'm in pain” not as derived from the observation of expectations and pains and their subsequent description, but as verbal expressions more on a par with how groans and smiles would express headaches and joys. In a similar way, Ryle claimed in *The Concept of Mind* that we should see first-personal utterances not as reports about certain frames of mind, but more as immediate disclosures or manifestations of those frames of mind: “If the lorry-driver asks urgently, ‘Which is the road to London?' he discloses his anxiety to find out, but he does not make an autobiographical or psychological pronouncement about it” (Ryle, 2000, p. 164). Anscombe, finally, likewise urged us not to confuse verbal expressions of intentions with conjectural estimations of future actions (Anscombe, 1963, p. 6)^2^.

This emphasis on the expressive dimension of first-personal statements, as opposed to considering them as the linguistic outcomes of a sort of auto-descriptive voyeuristic activity, certainly comes a long way in meeting the philosophical requirements we spelled out above. Both first-person authority and the first/third person asymmetry can now be explained by the obvious fact that I can hardly express the intentions, wants or headaches of others (which I, nevertheless, might observe in *their* expressions of them). Yet, furthermore, such an account can also be of considerable importance in light of some of the philosophical discussions that have traditionally surrounded Freud's—and in a different, though nonetheless equally decisive way, Lacan's—understanding and justification of “the unconscious”^3^. Let us briefly note in what sense this might be so.

One way of delineating Freud's discovery that seems to be beyond reasonable suspicion is that, as Laplanche and Pontalis put it, “psychoanalytic theory emerged from a refusal to define the psychical field in terms of consciousness” (Laplanche and Pontalis, 1988, p. 84). Very early on some philosophers thought this as paradoxical, but then it seemed it was above all Freud's recourse to the notion of “unconscious representation” that was considered paradoxical^4^. “Conscious representation,” on the other hand, appeared quite natural. Yet beyond this superficial dispute as to which psychical quality we want to assign to these representations or *Vorstellungen*, the reference to “representative ideas” or *cogitationes* was firmly established. Therefore, in contrast to, for example, Lacan's somewhat oblique way of defining the common denominator between Freud and Descartes in terms of a “subject of certainty” (Lacan, 1981, p. 35), there seems to be a much more straightforward reason why indeed “the Freudian field was possible only a certain time after the emergence of the Cartesian subject” (Lacan, 1981, p. 47). Obviously, the gesture of defining the “psychic field” in terms of representations, whether conscious or unconscious, and in its wake, postulating the mechanism of inner perception as their ever-faithful companion, present themselves as fine candidates for explaining this historical co-emergence. For if consciousness is conceived in terms of inner sense or self-perception, then it isn't too difficult to imagine the unconscious creeping in as soon as doubts were being raised as to the scope of this mechanism of mental illumination. As indeed Freud was about to propose in his famous article on *The Unconscious*:

In psychoanalysis there is no choice for us but to assert that mental processes are in themselves unconscious, and to liken the perception of them by means of consciousness to the perception of the external world by means of the sense-organs […]. Just as Kant warned us not to overlook the fact that our perceptions are subjectively conditioned and must not be regarded as identical with what is perceived though unknowable, so psychoanalysis warns us not to equate perceptions by means of consciousness with the unconscious mental processes which are their object. Like the physical, the psychical is not necessarily in reality what it appears to us to be. We shall be glad to learn, however, that the correction of internal perception will turn out not to offer such great difficulties as the correction of external perception – that internal objects are less unknowable than the external world (Freud, 1915, p. 171).

Although, of course, Freud's specific deployment of Kant as having advanced some sort of psychologistic reading of our faculty of knowledge is contentious, it is nevertheless clear in what way the justification of unconscious mentality is but a necessary consequence of this representational point of departure. If, indeed, our consciousness of mental contents is not merely the consequence of - as some contemporary phenomenologists have it—their “intransitive self-givenness”^5^, but rather, as Kant put it, a secondary *achievement*^6^, *viz*. the outcome of a synthetic act which both attends to and abstracts from immediate sense impressions, then the unconscious of such a (transcendental) consciousness lets itself be understood in basically two ways: not only as (i.) those latent mental processes that are unaccompanied by that act, but also (ii.) the anterior act itself as required for consciousness, the latter due to the fact that, as Kant put it, “I cannot know as an object that which I must presuppose in order to know any object” (Kant, 1999, A402)^7^. It is then no more surprising that this structural *rencontre manquée* between representational consciousness and its fugitive condition, a condition which *as condition* always both enables and hides itself in its product, was bound to serve as the conceptual figure through which psychoanalysis would take up its proper place as the latest and most faithful heir of the Cartesian cogito. This explains why, for Lacan, “the subject, the Cartesian subject” would function as “the presupposition of the unconscious” (Lacan, 2006, p. 712). This also explains why, for Zizek, it is only in psychoanalysis that “the forgotten obverse, the excessive, unacknowledged kernel of the cogito” is properly exposed (Zizek, 1999, p.2).

Yet, leaving these canonical philosophemes behind for now, what becomes of the unconscious when the reference to reflexive consciousness, inner sense and other related notions is rejected? Obviously, insofar as *these* are rejected in serving as a convincing account for first-person authority, so should their inverted offspring when it comes to accounting for unconscious phenomena. That is, if first-person authority is defined in terms of the capacity to *say* something about oneself without having to rely on conjectural inferences or observational evidence, then the limits to this authority should not be thought in terms of the possible *failure* of such third-personal procedures. Barring the latter, what marks an intention, fear or belief as unconscious is consequently not to be considered as something which I, *qua* epistemic subject, fail to acknowledge, but rather of something which I, *qua* speaker, seem unable to *do*.

In order to substantiate and further develop these introductory remarks, we will proceed in the following way: in the first part, Wittgenstein's grammatical remarks on the asymmetry between the first -and third-persons in psychological self-ascriptions are presented as an alternative to detectivist explanations according to which first-person authority is to be regarded as a matter of epistemic accomplishment. This expressivist account will set the stage for our second part, where it will be used to propose a non-epistemic analysis of the meaning of unconsciousness. Along the way, we will offer a critical discussion of both Freud's and Lacan's respective readings of the unconscious. Regarding the latter, we will reject the idea that the concept of the unconscious (i) necessitates the introduction of a (Cartesian) “subject of the unconscious” and (ii) could be deduced from the paradoxes of first-personal reference.

## Explaining first-person authority: from detection to expression

In our introduction, we tentatively characterized the phenomenon of first-person authority in terms of a certain subjective privilege that seems to be attached to a range of mental states, acts and attitudes. Now it is time to clarify what this idea precisely entails.

Firstly, the basic intuition which is captured by such notions as “authority” and “privilege” is that, all things being equal, I am usually the best person to ask if you want to know what I think, feel, intend, imagine and so on. Further classical examples of such a privileged set of mental states are those that are usually invoked to put an end to philosophical discussions on skepticism. Thus, according to Descartes, I can be mistaken whether I'm really taking a walk or writing a text, but not whether I believe this is so, or whether it seems to me that way, or judge it to be so. These typical *cogitatio*^8^ are all members of a larger class which Wittgenstein designated as “psychological verbs.” Amongst these psychological verbs, we find those which point toward experiences (“I see,” “I hear,” “I have a headache”), cognitive activities (“I think,” “I judge”), but also intentions (“I am going to …,” “I propose to …”) or acts of the will (“I've decided to,” “I want to”).

Secondly, our “subjective privilege” with regard to these psychological verbs basically consists in being exempt from the demands of verification or evidence that are usually attached to our ordinary judgments, which indeed can be contested, contradicted, denied or, at the very least, questioned as to their justification. This is the feature Wittgenstein had in mind with the formula “asymmetry of the first and third persons in the present indicative.” That is, if, for example, I say I have the beginnings of juvenile dementia, or a broken arm, then the reasons I would cite in order justify these claims would be the same kind of reasons as were I to say someone else had these medical conditions. Roughly, reasons pertaining to observation, testimony and inference. Hence, with respect to the question of justification, in these cases there is no asymmetry between, say, the first-personal statement “I lost my wallet” and its third-personal inversion “he lost his wallet:” both are justified by, for example, checking one's pocket. However, if I say I want to eat French fries or am contemplating Freud's *Future of an Illusion* or desiring to go on a holiday, that similarity to the third person vanishes. Clearly, there seems to be something strained, or perhaps even uncanny, about someone asking me to justify these claims (à la “So you believe you're contemplating Freud's writings. Ok, but how do you know?”). And if I were nonetheless tempted to respond to such queries in order to ward of any remaining misunderstandings, citing behavioral evidence or inferences to the best explanation to the effect that I am really contemplating would seem to make matters worse. In fact, as Wittgenstein points out, an important feature of the apparently incorrigible character of self-ascriptions deploying psychological verbs in the present indicative is that one cannot ask the subject *how* he knows. By contrast, the third-personal claim that someone else is contemplating Freud's writings is devoid of any such privileges and in principle susceptible to further demands for justification.

### Detectivism

So how are we to explain this remarkable capacity to talk in such a seemingly effortless and authoritative way about our concurrent thoughts, hopes and fears? Following Finkelstein's suggestion^9^, let us call *detectivist* any philosophical account that tries to explain first-person authority by invoking a special mode of *epistemic access* that allows the subject of these mental states to *know* about them. That is, detectivism takes the problem of first-person authority as actually being but one specific application of the more general epistemological question of knowledge—in this case “self-knowledge”—, and the problem is therefore, as befits such epistemological queries, of determining the origin and conditions of possibility of that knowledge. The implicit reasoning behind such a claim seems to be the following: in the same way as I would be unable to say of this particular object that it is a table when I do not know what it is for something to be a table, I would not be able to say, for example, that I want to watch *Better Call Saul* if I did not know what is for a mental state of mine to be one of wanting-to-watch that series^10^. The detectivist account of first-person authority therefore assumes a phenomenological position^11^ with regard to the conditions of self-ascription of first-person avowals: in order for a first-personal psychological avowal to have any reference, it is necessary to invoke a phenomenon of “experiential evidence” for, or “givenness” of, my mental states, in this case, the first-personal givenness of wanting-to-watch that series.

Now what it is that provides the subject with this evidence for her own mental states is something about which detectivists have held (and still hold) a variety of views.

A *first* traditional approach consists in postulating two *acts* in order to explain a subject's cognitive access to its mental states. The first act being quite simply whatever mental activity happens to occur in my consciousness, the second “reflexive” act being whatever epistemic activity that allows the subject to come to know about the first act by being “directed” upon or toward it. Here we encounter some illustrious notions like “introspection,” “inward observation,” “acquaintance,” “inner sense” or “higher order thoughts and representations,” but the precise nature (quasi-perceptual or representational) of these reflexive acts should not detain us. The essential thought is that such higher-order or two-act theories guarantee immediate knowledge of my first-order mental acts through the recourse to a distinct—logical, ontological, or temporal—further act, one that, moreover, can only be directed to my own mental states, not those of others. That is, I cannot “introspect” and hence know immediately whether the other is in pain or intending to go for a walk, I only have this privileged spectatorial position with regard to myself.

The objections and difficulties that such higher-order accounts have encountered have been repeatedly discussed elsewhere, so I will not recount them here *in extenso*^12^. Suffice for our present purposes to refer to two of those that have figured most prominent amongst them: on the one hand, ever since Hobbes, Leibniz or Brentano, it has been noted that two-act theories always lead to an infinite regress which can only be blocked by positing anterior unconscious mental acts^13^. On the other hand, the fact that self-knowledge is granted such unusual epistemological features as immediacy, infallibility, immunity to error through misidentification, and so on, makes it hard to understand how any quasi-perceptual or representational mechanism would ever be able to explain these. For it seems to be part of our language games that talk about perception and representation at least implies the possibility of misperception and misrepresentation. Yet insofar as the latter are already excluded by the very definition of the detectivist explanandum, so should their extraordinary counterparts if we want to keep using our concepts of perception and representation in any meaningful way^14^.

A *second* approach, most commonly associated with phenomenological writers such as Sartre or Husserl, tries to explain the special knowledge I have of my mental acts, while evading the regress objection, by simply stating that such psychological verbs like thinking, perceiving or doubting are “acts of consciousness.” In keeping with Brentano's definition of mental phenomena, such acts of consciousness are said to be *intentional*, i.e., they are acts through which I am immediately conscious *of something*; differently put, they are what allows me to relate to a world that is transcendent to my consciousness. Perception, for example, is that mode of consciousness through which a perceptual object appears; imagination, that mode of consciousness through which something is given as imagined, thinking, that by which something is thought, … Therefore, on this account, and in contrast to two-act theories, one does not say that my intention to go on a holiday is something I perceive or represent by way of a further act, but rather, that my intention is a “consciousness” of a future holiday in the way of intending it. Similarly, one does not say that my desire for a bloody mary is something I apperceive as a disinterested spectator, but that desire is a mode of consciousness that puts me in a relation to a bloody mary in order for the latter to appear desirable. One has to distinguish, as Husserl (1976, pp. 303-4) put it, the cogitatum from its cogito, the intentional object as it is intended (*im Wie seiner Bestimmtheiten*) from the intentional act through which it is intended (*im Wie seiner Gegebenheitsweisen*). It is, in other words, through the very transitive or “thetic” consciousness of, say, a burning house as perceived or imagined, that I am immediately alerted to the intransitive or “non-thetic” act of perception or imagination. Importantly, it is only because the act through which I am consciously related to objects is itself also “experienced,” *viz*. that there is something “it-is-like” to perceive, believe, think and intend, that I am subsequently able to report on that act and its intentional object whenever I'm asked. To put it in the typical phenomenological neologisms for denoting this kind of intransitivity, such pre-reflexive experiences are said to be “intrinsically self-revealing,” “self-disclosing,” “pre-reflectively self-given,” “self-appearing” or “self-manifesting” (for these ideas, see Henry (1973)). Coining another neologism appropriate to our discussion, we can say that such acts of consciousness are “self-detectifying”: that is, it is in their very nature to procure their own evidence.

Yet, apart from the strange observation that in order to make sense of such one-level accounts, we have to invoke what appears to be *sui generis* epistemological procedures and associated philosophical vocabulary, it is clear that the proposal's *prima facie* plausibility derives from the decision to extend the class of experience verbs so as to include acts like thinking, judging or remembering. Apparently, all these different psychological verbs now become “acts of consciousness,” and therefore, insofar as my ability to report on them is explained by their “self-revealing” character, must have a distinctive phenomenology about them. Yet, believing that it is about to rain or remembering to take out the garbage is hardly to have experienced something in the mode of thinking or remembering, and it is highly questionable whether there even is something like an experiential quality of the act of intending to read the newspaper. But even in the case of proper experience verbs like “feeling ill” or “sensing a pain in my shoulder,” it is difficult to conceive in what way my self-ascriptions of illness or pain could amount to any sort of epistemic achievement. For does it make sense to say that “I know I'm not feeling quite well today” because I have a pre-reflexive consciousness of my current misery? Or, indeed, more generally, to say that “I know” these troublesome sensations at all?^15^ Obviously, epistemic operators like “I know that p” or “I know whether p” only make sense if they can be used in contrastive conjunction with their negations or modifications like “I don't know that p,” “I doubt whether p” or “I wonder if p.” Whereas in the third-person case, I can indeed be ignorant or remain in doubt about someone else's feeling of nausea, similar expressions in the first-person present tense like “I wonder if I have a headache,” “I have a pain in my back, but I'm not sure,” …, are strictly speaking meaningless. Yet they are not meaningless because, to paraphrase Sartre, we are dealing with “a cogito that retains its rights even with psychopaths” (Sartre, 2004a, p. 148), or with the ineliminable occurrence of a consciousness unable to forget itself, but rather because there are no epistemic rights to retain, nor any business of forgetting or remembering involved in the first person present employment of such psychological verbs.

### Wittgenstein's expressivism

Now the latter conclusion might, for some, already count as a *reductio ad absurdum* of the critique of detectivism just presented. To deny psychological self-ascriptions any epistemic purchase seems to amount to denying the phenomenon of first-person authority itself. That is, if it can't be said that I know whether I have a headache or believe that it is raining, why then should I be credited with any kind of authority on the matter? Additionally, anticipating our discussion of unconscious mental states, if talk about knowledge is denied for those beliefs and desires we are seemingly in a position to avow without any hesitation, then it seems to be a direct consequence of such a non-epistemic approach that talk about beliefs and desires that do not share these first-personal asymmetries is equally ruled out in advance.

As pointed out in our introduction, one way of addressing this problem which arises from Wittgenstein's later work, begins with the suggestion that our self-ascriptions owe their status, which is so apparently authoritative, to the simple fact that they are not, after all, *reports* of current psychological states, but rather count as *expressions* of the states they only appear to describe on a superficial consideration of their surface grammar. In the case of sensation-language, Wittgenstein puts this point as follows:

It is not, of course, that I identify my sensation by means of criteria; it is, rather, that I use the same expression. But it is not as if the language-game *ends* with this; it begins with it.

But doesn't it begin with the sensation – which I describe? – Perhaps this word “describe” tricks us here. I say “I describe my state of mind” and “I describe my room”. One needs to call to mind the differences between the language-games (Wittgenstein, 2009, §290).

What are “the differences between the language-games” of describing one's room and describing one's state of mind Wittgenstein is hinting at in this quote? An important point seems related to—if we can put it that way—the “temporal logic” of the descriptive ascription. Wittgenstein appears to be intent on denying that in the case of, for example, saying that I am in pain, I should begin with the examination of my pain-sensation (through, say, identifying my headache *as* a headache by means of headache-criteria) before proceeding to the self-ascription; whereas, in the case of describing my room, it is of course indispensable that I should have observed things and looked it over before engaging in my description. In the latter case, the description comes at the *end* of the language game—after I have identified whether the room is spacious or contains a *chaise longue*. Moreover, this identification is what in fact *justifies* my descriptive assertions. Yet, if Wittgenstein denies that this is what we ordinarily do when giving one's thought on some philosophical matter or complaining about a pain in one's back, then what is it that justifies these first-personal ascriptions? And what does it mean to say that in this kind of language game such ascriptions come *first*, rather than at the end of some justificatory activity? To continue the analogy with the room-description, one might think, for example, that to begin with the ascription would actually amount to describing one's room without looking at it first, that is to say, to simply engage in some highly speculative endeavor. And since, in fact, Wittgenstein abrogates epistemic justifiers in the case of psychological self-ascriptions, this seems to leave us with the following option: to construe such talk as being merely the result of—to use Dennett's expression (Dennett, 1991, p. 67)—some “impromptu theorizing”. However, that this is *not* the conclusion Wittgenstein wants to draw from his critique of inner observation or any similar detection mechanism, becomes clear in the following passage:

“When I say ‘I am in pain' I am at any rate justified *before myself*.” – What does that mean? Does it mean: “If someone else could know what I am calling ‘pain,' he would admit that I was using the word correctly”?

To use a word without a justification does not mean to use it wrongfully (Wittgenstein, 2009, §289).

So, according to Wittgenstein, it is not because I am not “justified before myself” in saying that I'm in pain, that I am therefore *unjustified* when resorting to this pain-talk in the sense of making a mistake or simply talking nonsense. Such a confusion will remain as long as we do not reject “the grammar which tends to force itself on us here” (Wittgenstein, 2009, §304), which is precisely the descriptive grammar that is premised on the model of “object and name” (Wittgenstein, 2009, §293) as in the case of the description of my room. To rid oneself of the idea that, in order to avoid skeptical worries about our “folk-psychological” practices, authoritative psychological self-ascriptions of beliefs and sensations *must* be grounded in evidence roughly in the way that the observation of my room supports my subsequent descriptive assertions, we have to question the assumption that first-personal avowals function as *reports* about psychological facts the speaker has previously learned or ascertained. This is the guiding thought behind Wittgenstein's following remarks:

The paradox disappears only if we make a radical break with the idea that language always functions in one way, always serves the same purpose: to convey thoughts – which may be about houses, pains, good and evil, or whatever (Wittgenstein, 2009, §304).

When someone says “I hope he'll come”, is this a *report* [*Bericht*] about his state of mind, or a *manifestation* [*Äußerung*] of his hope? – I may, for example, say it to myself. And surely I am not giving myself a report (Wittgenstein, 2009, §585).

Here we retrieve the earlier suggestion that psychological utterances are primarily *manifestations* or *expressions* of the very states they self-ascribe, rather than secondary thoughts or beliefs about these states uttered to inform the listener about whatever psychological fact it was I had previously ascertained. My utterance of “I believe (hope, desire, intend, …) that *p'* hence expresses, rather than reports, my belief (hope, desire, intention, …) that *p*. Therefore, utterances like “I am in pain” or “that really hurts!” are not pain-reports on a par with weather-reports, but, like my moans or cries, manifestations of my pain; similarly, the utterance “I want to go out” does not inform someone about some planning experience, but voices, depending on the context in which it is said, my desire to go out or my agreement to your proposal; finally, ardent exclamations like “I love you” or “I really hate your guts” do not communicate subjective facts to which you may or may not agree or proceed to inquire some further information, but should be more properly considered as verbal equivalents of bringing flowers and throwing plates in your direction.

Now, to conclude this section, what is the philosophical import of Wittgenstein's expressivism with regard to our discussion of first-person authority and how precisely does it differ from the detectivist solutions earlier discussed?

First of all, despite some other difficulties it has encountered^16^, expressivism has one striking advantage when compared to other accounts of first-person authority: the reason why, indeed, I am usually the best person to ask if you want to know what I believe or desire, is that, on an expressivist account, my self-ascriptions ordinarily count as manifestations of those beliefs and desires, roughly in the same way as driving a car shows my ability to do so, or as blushing reveals my embarrassment. This means that, contrary to detectivist accounts, there is then *no* epistemological question to be answered about how I am able to avow my beliefs, fears and sensations, just as my “ability” to laugh with your joke requires no preliminary introspective investigation nor realizes any epistemic accomplishment. And since my mental state self-ascriptions are typically not reports or descriptions of these mental states, that is, since they do not involve subjective *judgments* about the presence of these mental states, solicitations for further proof or evidence should not be dismissed on the grounds of being overly scrupulous superfluities, but merely as betraying a fundamental misunderstanding of the expressive character of my linguistic performance.

Secondly, starting from this expressivist appraisal of psychological avowals, we can also begin to see in what way expressivism allows for an elegant solution of the first-third person asymmetry in mental state ascriptions that avoids some of the pitfalls detectivist accounts have encountered. That is, expressivism should be understood as claim about what is special to the use of psychological self-ascriptions in contrast with a wide array of other uses. But the contrast to be effected is not between a first-personal intimate, direct epistemic relation, by comparison to which third-personal inferences from behavior, for example, are indirect and less secure; rather, the asymmetry rests entirely on the difference between simple present tense expressive uses and all other employments of psychological terms. As Wittgenstein remarks in the *Blue Book*:

The difference between the propositions “I have pain” and “he has pain” is not that of “L.W. has pain” and “Smith has pain”. Rather, it corresponds to the difference between moaning and saying that someone moans (Wittgenstein, 1969, p. 68).

So what is distinctive about an avowal like “I have pain” in contrast to its third-personal inversion “he has pain” is that in the former case, the avowal is issued by the very person who is said to be in pain, at the very time she is in pain, in the course of expressing her pain. Obviously, this is something I am in a unique position to do: only *I* can express or give voice to my own present states of mind, and it is only states of *my* mind that I can express. By contrast, my ascriptions of mental states to others, e.g., saying that someone else is in pain, will typically count as descriptions or reports of these states of mind as expressed in their (linguistic) behavior. In that case, my assertions can serve to express my beliefs about whether or not “he is in pain,” but thereby I do not, of course, express his pain, and furthermore, I ought to be able to provide some reasons for my belief (for example, because I saw he was in pain, or because he told me so). Furthermore, this also explains why our first-person privilege extends only to *some* aspects of our mental lives and, thus, why the asymmetry between first -and third-person ascriptions is not reducible to the distinction between my psychological states and those of the other. Since first-person authority is restricted to those avowals which express my mental states in self-ascriptions of them, past-tense ascriptions (“I felt really sad while watching that movie”), future-tense ascriptions (“I will feel better after going for a walk”) or self-attributions of psychological dispositions (“I'm a hopeless neurotic”) will fall on the third-personal side of the asymmetry because my assertions about these matters will not express those states themselves^17^.

Finally, if, as we have argued, this asymmetry is not the consequence of any supposed epistemic asymmetry, then some of the skeptical worries that have continued to resurface with regard to the phenomenon of first-person privilege permit for a different response. To take but one famous example drawn from the philosophy of cognitive neuroscience: from the deficiency of the Cartesian theater and its associated spectatorial accomplice in serving as a convincing account of first-person authority, Dennett famously argued for a view on first-personal avowals that regards them as “theorist's fictions” (Dennett, 1991, p. 98), that is, as provisional beliefs about mental items whose putative reality will be corroborated (or not) depending on future developments in neuroscientific research. As Dennett puts it:

People undoubtedly do believe they have mental images, pain, perceptual experience and all the rest, and these facts – the facts about what people believe, and report when they express their beliefs – are phenomena any scientific theory of mind must account for (Dennett, 1991).

Here we see clearly how staunch materialists like Dennett, despite rejecting a metaphysically suspect account of what makes the phenomenon of first-person authority distinctive, still retain the essential idea that sustains the detectivist picture of psychological avowals where the latter, as we have seen, always serve to express *beliefs* about mental life, rather than mental life itself^18^. Yet, it is here that the explanatory fecundity of Wittgenstein's proposal most clearly shows itself: in the very place where materialist detectivists locate a class of psychological entities in order to reduce or eliminate depending on future evidence, Wittgenstein points toward a distinctive employment of psychological vocabulary in relation to which the usual questions of justification and evidence quite simply do not arise. However, in making this distinction, it is clear that Wittgenstein, in contrast to philosophers like Dennett, does not mean to draw our attention to any first-person epistemic short-coming, nor means to relegate psychological avowals to the domain of fiction; the claim is rather that our concept of knowledge (or, for that matter, fiction) cannot handle the relation between psychological avowals and mental life to begin with, and consequently, that the deficiency of self-knowledge should not be invoked to raise skeptical arguments against our ordinary practice of speaking our minds.

## Expression and the “as-if” of the unconscious

As suggested in our introduction, apart from its philosophical potential to offer a non-Cartesian solution for the asymmetry between first- and third-person psychological ascriptions, Wittgenstein's expressivist reading of the kind of authority involved in first-person avowals also permits for a different take on what is or should be one of the major preoccupations of psychoanalytic theory: i.e. to offer a perspicuous account of “unconscious subjectivity.”

### Unconscious: adverbial vs. substantial

However, in setting up the problem in this way, that is, by tacitly assuming that a philosophical reading of what it actually means to talk about oneself in an authoritative or non-authoritative way could have significant implications for psychoanalytic theory, it might be objected we are already betraying one of the fundamental tenets of the Freudian approach. And in a certain way this is correct: what is indeed already excluded from further consideration by taking this angle of approach is the reference to “the unconscious” which results from the grammatical transformation of adverbial qualifications such as “he unconsciously believed *p*” or “he had the unconscious desire to *p*,” into a substantive employment of the term in phrases like “his unconscious believed *p*” or “this might have something to do with my unconscious.” That is, there might be a perfectly intelligible reason to speak of someone as, for example, harboring an unconscious desire to please his analyst, or as manifesting an unconscious belief in the omnipotence of thoughts, and to say that, as Freud brilliantly put it (Freud, 1905, p. 77), even though in such cases “his lips are silent,” he nonetheless “chatters with his finger-tips.” And one (relatively) straightforward way of justifying such adverbial locutions consists in bringing out *why* the reference to “unconscious” matters in such descriptions, or conversely, whether it would make a considerable *difference* if the possibility of using such qualifications would henceforth be denied (say, for example, if instead of using the term “unconscious,” we would be obliged to resort to expressions like “unknowingly” or “inadvertently”).

Yet, an entirely different philosophical problem seems to arise from the moment we leave this descriptive domain of things we might have done or believed unconsciously, and suddenly decide to talk about beliefs, desires, processes or even “subjects” *of* the unconscious. As Wittgenstein put it:

Imagine a language in which, instead of saying ‘I found nobody in the room,', one said ‘I found Mr. Nobody in the room'. Imagine the philosophical problems which would arise out of such a convention. Some philosophers brought up in this language would probably feel that they didn't like the similarity of the expressions ‘Mr. Nobody' and ‘Mr. Smith' (Wittgenstein, 1969, p. 69).

And indeed, the sort of discussions that emerge from this substantialist transformation, including the lingering conceptual *unbehagen* Wittgenstein was hinting at, are sufficiently familiar in psychoanalytic circles: here, of course, we encounter the classical and intractable debates gathered under the heading of “the reality of the unconscious” which have animated a good deal of the philosophical literature. Since the concept is now no longer invoked in order to describe but to *explain* certain phenomena, “the unconscious” is henceforth taken to refer to some kind of intangible entity—depending on one's humanistic sensitivities and scientific aspirations, a second mind or a more impersonal variety—able to exert a more or less causal influence on my current behavior, secretly operating from what Freud famously called, as a sort of backstage-addendum to the Cartesian theatre, “der andere Schauplatz.” Closely following from this conception are the typical queries probing its ontological credentials, i.e. whether or not such a thing exists and if so, to put it in Heideggerian terms, what “type of existence” or “manner of being” we are dealing with (is it a reified thing, a slightly less reified event, a new regulative-transcendental category, …); what kind of “stuff” it is made of (drive representatives, mnemic traces, psychical “matter,” pure intensities?), by which kind of laws, modes of production or mechanisms (condensation, displacement, metaphor/metonymy, …) it might be able to produce its effects and what sort of “causality” (“psychical” or the more progressive “structural” sort) is possibly involved here. So, without going into much further detail, it should be clear that insofar as this is indeed the meaning one wishes to assign to “unconscious,” the questions that would follow from such a substantial employment would obviously exceed the scope of our analysis, for in that case the justification of the concept coincides, as it should, with the (scientific) demonstration of its explanatory potential.

However, as has been noted by numerous commentators, there is on the other hand ample reason to resist reducing the discussion on the logical status of the unconscious to this substantialist locution, not merely because Freud's arguments for introducing such a notion are essentially vacuous, but also because that employment has been repeatedly denounced—most notably by Lacan—within psychoanalytic theory itself. Both issues here—i.e., the reasons for both the explanatory vacuity of the unconscious and Lacan's dissatisfaction with it—can be put in sharper focus by revisiting the well-known arguments by which, very early on, Sartre rejected the Freudian unconscious as a possible explanation for the phenomena of “bad faith” and self-deception. Here's how Sartre famously described the problem:

By the distinction between the “id” and the “ego”, Freud has cut the psychic whole into two. I *am* the ego but I *am* not the id. I hold no privileged relation to my unconscious psyche. […] Thus psychoanalysis substitutes for the notion of bad faith, the idea of a lie without a liar; it allows me to understand how it is possible for me to be lied to without lying to myself since it places me in the same relation to myself that the Other is in respect to me; it replaces the duality of the deceiver and the deceived, the essential condition of the lie, by that of the “id” and the “ego”. It introduces into my subjectivity the deepest intersubjective structure of the Mit-sein. Can this explanation satisfy us? (Sartre, 2003, p. 51).

And the reason why, in short, according to Sartre, the latter rhetorical question must be answered negatively is not so much due to, as it is often portrayed, phenomenological arguments invoking the insulated appeal to “lived experience,” but rather thoroughly *conceptual*^19^. More precisely, that whenever the psychoanalytic explanation is indeed fully and explicitly set out, we find that the introduction of the substantial use of “the unconscious” as something that “lies” or “deceives” in my place merely relocates the descriptive facts of a person's actions and intentions at a putatively sub-personal level. It is then only through the optical-illusory device of partitive sub-personal re-description that the unconscious can be made to *seem* as a genuine explanation; what has been offered in reality is merely a trivial reification of the adverbial meaning of unconscious, one which, moreover, accords quite well with the self-deceiver's own self-misrepresentation (see Gardner, 1993, pp. 41–58)^20^. And here, furthermore, we also encounter the main source for Lacan's misgivings with respect to this spontaneous tendency to “reify” or “hypostatize” the unconscious. As already evident from Sartre's remarks, there is indeed, apart from this conceptual confusion, a further compelling reason why such a view cannot be endorsed by psychoanalytic theory. For as soon as the concept is understood in this way, a comforting picture imposes itself where the unconscious is situated, so to speak, on the outside, and we ourselves, as “healthy ego's” or unsuspected spectators, on the inside, thereby able to occupy a transcendent position toward an unconscious which becomes nothing more than, as Sartre put it, an “experimental idea” (Sartre, 2003, p. 51). Yet, as Lacan argues in a Spinozistic way, the unconscious is not what we *qua* conscious egos are set over and against, nor is the Freudian *Spaltung* reducible to a mere shifting from one center of (irrational) agency to another; rather, according to Lacan, this movement of displacement or self-differing is itself precisely what we, qua subjects, *are*^21^.

### The unconscious structured as a cogito

It will take us a few detours to elucidate such an elusive idea which, as it stands, is neither of immediate relevance to our discussion on first-person authority, nor particularly helpful in answering the question we set out in the beginning. There we asked quite simply what, if anything, we are trying to get hold of by describing someone's belief, pleasure or intention as unconscious. And the outcome of our discussion so far has been mainly negative: nothing in particular is gained through the shift from the adverbial to the substantive use of unconscious that was not already left open in the former, nothing but, perhaps, a momentary illusion of sur-plus explanatory value.

Now it is often suggested that the philosophical relevance of a figure like Lacan stems precisely from the fact that he, in contrast to some of Freud's perhaps rather unfortunate formulations, decisively “de-substantializes” the unconscious. Although it is seldom made explicit why, in all, this is seen as a conceptual progression in the first place—for example, because of the absurdity of second mind-conceptions, or the daunting prospect of having to furnish a convincing “de-homunculized” alternative, … -, it is nonetheless generally agreed that Lacan's putative achievement more specifically consists in the very *way* he proposes to de-substantialize the unconscious, that is, by explicitly engaging with what, at first blush, seemed directly opposed to it, namely, the modern problematic of the subject as formulated by Descartes^22^. And indeed, compared to Freud's rather indifferent attitude toward whatever it was Descartes hoped to establish by means of his hyperbolic diligence^23^, it is certainly true that Lacan lucidly exploited the ever productive paradoxes which result from the philosophical decision to conceive the apparent certainty of the Cartesian *cogitata* as grounded in a reflexive, transitive relation to oneself. That is to say, precisely that sort of self-reflexive relation on account of which Sartre believed to protect translucent consciousness from encountering any unconscious opacity among one of its self-negating moments, is now deployed in order to expose its “inner limit” or “hidden truth.” In sum, instead of an unconscious which simply reduces to an anonymous other whilst leaving the authority of the Cartesian cogito intact, Lacan promotes the latter figure as the most suitable *persona* from which the meaning of “unconscious” could be deduced; instead of a determining substance as opposed to a reflexive subject, Lacan proposes a “subversive” unconscious reflexivity^24^, or, to condense the foregoing permutations, a “subject of the unconscious”:

in the term subject … I am not designating the living substratum needed by this phenomenon of the subject, nor any sort of substance, nor any being possessing knowledge in his pathos, his suffering, whether primal or secondary, nor even some incarnated logos, but the Cartesian subject, who appears at the moment when doubt is recognized as certainty – except that, through my approach, the bases of this subject prove to be wider, but, at the same time much more amenable to the certainty that eludes it. This is what the unconscious is (Lacan, 1981, p. 126).

Now, although it may seem that this particular psychoanalytic version of the cogito constitutes a significant break with a long tradition of exegetical commentary dedicated to determining the precise meaning of Descartes' *fundamentum inconcussum*, it should be apparent however, already from this short quote, that what we are dealing with is actually but one further variation within that same tradition. For when understood as a cogent response to our initial question—i.e., what does it mean for someone's belief (desire, intention, …) to be unconscious?—, then already one part of Lacan's answer cannot but appear as remarkable. That is, assuming that one horn of that question consists in simply asking “*whose* unconscious belief are you talking about?,” why is it, after all, that we are suddenly referred to the austere figure of the “Cartesian subject”? Whence this idea that the subject that truly matters to psychoanalysis is, as Lacan (2006, pp. 870–872) emphasizes, not to be confused with “the suffering subject,” “the subject in relation to his body” or “the subject of love”? Is it, for example, somehow an intrinsic part of psychoanalytic experience that those who participate in that strange *folie-à-deux* turn out to be, on closer inspection, “dehumanized” and “disembodied” subjects devoid of any empirical individuality, strangely akin to those selves of solipsism of whom Wittgenstein (2002, §5.64) aptly remarked that, eventually, they collapse into “points without extension”?

It seems altogether more likely that we have to locate the principal source of this idea *elsewhere*, and more in particular, in those philosophical readings which, as Anscombe pointed out in her classical article on the first person (Anscombe, 1981a), all have tended, in their own distinctive ways, to take the “I” of Descartes famous formula as a referring expression (Anscombe, 1981a, p. 22). In terms of our general discussion, the issue here is closely related to the earlier discussed asymmetry between the first and third persons with respect to psychological verbs, only this time focused on the particular role or function fulfilled by the word “I.” As is well known, in the *Blue Book*, Wittgenstein (1969, pp. 66–67) proposes a distinction between two different employments of the first-personal pronoun: on the one hand, those instances where the words “I,” “me” and “mine” serve the speaker in talking about himself as an object (e.g., his body) and to attribute some property to this object on the basis of an observation. Examples of this kind of use are: “My arm is broken,” “I have grown six inches,” “The wind blows my hair about,” … Since such statements are used to convey descriptive information resulting from an observation, they are constructed like every other assertion regarding an individual: they comprise a predicate (whatever it was I did observe) and a subject (the object on account of which I did the observation). Accordingly, there are two ways in which such “I-as-object”-statements could be susceptible to errors of identification. Be it on the side of the predicate, because, for instance, my arm turned out not to be broken after all; or on the side of the subject, that is, the arm is indeed broken, but it's not mine. The latter error is due to the fact that I have confused one person with another, namely myself, and its possibility indicates that what is involved in this type of employment is the reference to and recognition of a particular person. By contrast, the second type of employment, where the speaker appears “as subject,” excludes any sort of error with regard to the identity of the person involved: when I say “I see someone approaching” or “I believe it will rain,” I might be wrong in the sense that in fact there's no one there or because my forecast got it wrong, but certainly not because the one who was seeing or believing turned out to be someone else after all. Or, as Anscombe illustrates (Anscombe, 1981a, p. 30), when, during a dinner, a bishop lays down his hand on the lady's knee, he could try to flee the embarrassing moment by claiming that he took the lady's knee for his own, but, in any case, *not* by conceding that he mistook himself for the lady in laying down his hand.

Now this fundamental distinction with respect to the different ways we speak about ourselves has often been translated in such a way as to make it congenial to a traditional theory of the subject. For it is clear that, thus presented, the opposition between these two different kinds of employment of the first-personal pronoun not only seems to correspond with the distinction between physical properties (having a broken arm, measuring a certain height, …) and psychological properties (seeing, believing, …), but also between two types of “subjects” or bearers of these properties. That is, between someone who can be identified and referred to in a way that is symmetrical for the first and third persons, i.e. the objective-empirical person, and someone who can only be recognized and referred to in a special first-personal way and who is, *for that very reason*, also a very special person, i.e., a Cartesian subject or ego. This last point is particularly important because it enables us to see not only why, as Lacan rightly emphasizes (cf. supra), such a subject defined in terms of an exclusive first-personal reference cannot have anything to do with the concept of a concrete human being, but also why it is precisely on the basis of a certain radicalization of this referential thesis that Lacan wants to establish his idea of the unconscious (cf. infra).

To render this conspicuous, we have to bring out why the Cartesian self is not so much the idiosyncratic outcome of Descartes' purifying reductions, but rather of the philosophical attempt to satisfy two basically incompatible conditions in explaining the seemingly guaranteed success of the “I-as-subject”-use. That is, the Cartesian explanation presupposes, on the one hand, (i) that “I,” in examples of its use as subject (e.g., “I believe it will rain,” “I doubt whether I exist,” …), *like* uses of other subject-terms in grammatically similar ascriptions (e.g., “I have a bump on my forehead,” “my eyes are blue,” “my name is René,” …), purport to refer to an object of which something is to be predicated. This in turn presupposes that such self-ascriptions must rely on some form of access to, or recognition of, the referent of “I.” Yet, on the other hand, (ii) the distinctiveness of this kind of “I”-ascription relative to, for example, the present tense use of “I-as-object,” other-ascriptions or past-and-future tense ascriptions, is held to be a matter of epistemic security: *unlike* these other referring expressions, the use of “I” as subject is immune to error through misidentification, which is to say that in this case its referential success is thought to be guaranteed. However, from the effort to *combine* these two conditions, i.e., semantic continuity and epistemic asymmetry, in explaining what is specific to the employment of the “I-as-subject,” it necessarily follows (iii) that the subject who is referred to in such ascriptions cannot be individuated or identified on the basis of *any* identity criteria, since, as a matter of conceptual or logical necessity, talk about reference and identification implies the possibility of their failure or misapplication. Hence the idea, pointed out by Lacan and several other Descartes interpreters, that the ego which gloriously survives the increasingly stringent stages in the skeptical cogito-procedure, has nothing in common whatsoever with the historical-empirical figure of Descartes, nor with any conceivable personal identity: such an ego is said to be “purified” from all possible determinations, like Pascal's “ego without qualities,” Kant's formal “I-think” or Husserl's “reine Ich.”

Yet, leaving the philosophical genealogy of the cogito aside for a moment, this also means that the prospect of getting a satisfactory answer to our initial question suddenly appears to be rather dim. Since if that answer, at least for the part that interest us here, consists in claiming that the subject we are dealing with is an existence without determination, an empty form deprived of all substance and content, in short: a thinking or doubting in general, then it is not only that subject which appears as “void,” but, it seems, so does our whole questioning. For imagine, for example, what such an answer would amount to for a psychoanalyst who wants to distinguish between two of his patients, or perhaps between himself and his patient, while being forced to proceed on the basis of the Cartesian clue “a thinker or thinking in general.” It is clear that even a simple task like this would quickly reveal itself as being utterly hopeless. Whatever other theoretical need it is supposed to fulfill, the referential description “a subject in general” is of course completely useless in distinguishing one subject from another, for all identity criteria which would allow us to answer that question have been expelled, but the theoretician of the subject, whether he wants it to be conscious or unconscious, has failed to give us a proper alternative. Being a “subject in general,” therefore, is perhaps to be a subject in a contrived philosophical sense, but not the analyst, not the patient, nor anyone else. As Descombes put it (Descombes, 2014, p. 68, our translation), it is rather like having an “address in general,” that is to say, of living somewhere, but nowhere in particular. And it should be clear that this is not an “ontological” invitation to henceforth distinguish between two types of addresses, those that are particular and those that are general, but rather to question how we ended up with such non-localizable entities in the first place.

### The cogito structured as an unconscious

Already one part of that answer we tried to elucidate above: in trying to account for the difference between the first and third-person uses of the first-personal pronoun, the Cartesian subject emerged as a sort of unstable “compromise formation” resulting from the attempt to straddle both sides of the following fence at once. On the one hand, aiming to preserve semantic continuity, the expression “I,” like all others referential expressions, is considered to be the term through which a speaker or thinker refers to himself. Yet, on the other hand, aiming to preserve epistemic asymmetry, such reference cannot have any of the ordinary features normally attached to this kind of operation: as Anscombe remarks, “I” is supposed to have “sure-fire”-reference (Anscombe, 1981a, p. 30), a remarkable quality which in turn seems to contradict its professed semantical function. Furthermore, unwilling to confront this precarious dilemma in which epistemic asymmetry undermines referential symmetry and vice versa, the Cartesian is moved to advance an extra-ordinary referent corresponding to his idea of extra-ordinary reference: this is the Cartesian subject or ego.

We pointed out how this referential interpretation of the “I” seems to saddle us with a mysterious entity which, despite remaining certain in its doubtfulness, is nonetheless entirely useless when it comes to answering even the most basic identity questions. So, in so far as the issue here does not merely pertain to the particularities of the alleged outcome of Descartes' Second Meditation, but, as Lacan suggests, equally concerns the everyday practice of psychoanalysis, then at least one clear undesirable consequence of embracing this figure as the principal term of reference in psychoanalytic theory is that the dialogical situation between an analyst and his patient would inevitably collapse into an unmanageable state of identity confusion.

Be that as it may, this still leaves us with the task of unravelling in what sense Lacan's peculiar deduction of the meaning of ‘unconscious' from the formula of the Cartesian cogito is to be understood. For it is clear, of course, that Lacan is not in the Cartesian business of proving he has a soul, nor intent on shoring up any indubitable entity that could come to function as an initial bedrock for subsequent world-involving epistemological deliberations. The intention here is rather to denounce what he holds to be the “initial error of philosophy” (Lacan, 1987, p. 107)^25^, which, moreover, apparently consists in the ever-renewed attempt to reduce the difference between “subject and consciousness” (Lacan, 1987, p. 106):

At a crucial point of the Cartesian *askesis*, […] consciousness and subject coincide. It is holding that privileged moment as exhaustive of the subject which is misleading—making of it the pure category that the presence of a gaze (as a mode of opaqueness within the visible) would come to make flesh with its vision […]. It is, on the contrary, at that moment of coincidence itself, in so far as it is grasped by reflection, that I intend to mark the site through which psychoanalytic experience makes its entrance. At simply being sustained within time, the subject of the “I think” reveals what it is: the being of a fall. I am that which thinks “Therefore I am” […] noting that the “therefore,” the causal stroke, divides inaugurally the “I am” of existence from the “I am” of meaning (Lacan, 1987, pp. 106–107).

Yet, here already, and despite the air of radical “subversion” which is supposed to transpire throughout these lines, it is remarkable that Lacan actually *shares* the detectivist gesture of invoking “consciousness” as a special medium of epistemic access (“the presence of a gaze,” “grasped by reflection,” …) in explaining what allows Descartes, or any other person for that matter, to declare that “I think (‘therefore I am')”. Secondly, we also encounter the referentialist framework within which such detectivist ideas are able to flourish: saying that “I think (‘therefore I am')” is, on that account, to be considered as a linguistic act through which I refer to, and identify with, a person (i.e., “myself,” or, to keep with Lacan's third-personal inversion “my *self*”) under a particular description^26^, in this case “I am that which thinks ‘therefore I am'.” So, revealingly, it is in any case *not* this traditional account of first-personal reference which might be critically questioned and identified as philosophy's “initial error,” but rather that of holding this detectivist moment as “exhaustive of the subject” (Lacan, 1987, p. 106). Or, differently put, not the idea that I, in speaking about myself—whether this means saying things like “I think I exist,” or less dramatically, “I'm not feeling too well,” “I believe it will rain,” …—have to engage in a sort of mysterious auto-reflexive procedure at the end of which, on the one hand, the term “I” serves to designate or name the person who is currently speaking^27^, and its verbal complements (“not feeling too well,” “believing it will rain”), on the other hand, function as descriptive predicates filling out the first-personal proposition, but the idea that this procedure is actually able to attain its self-reflexive intention.

And indeed, doubts as regards the feasibility of this kind of auto-referential operation are easily generated on account of a famous paradox to which such views unavoidably lead. For if speaking about myself in the first-person is represented as yet another version of third-personal reference, that is, if, in saying “I know I exist” or “I believe it will rain,” I refer to myself as someone who knows or believes thus, to *who* then exactly does this reference apply? The subject, for example, who, in turning his attention to himself, knows that he believes he exists, does he also believe he exists? Or, to take a more clinically relevant example, the patient who, during his weekly session with the analyst, suddenly avows his intention to change his life, does he also want to change his life? Moreover, given this idea of first-personal reference, could he even be imagined as ever being able to answer that question? Obviously not, for if we are to follow Lacan's reconstruction of the cogito, such a patient is forever condemned to occupy a spectral position of perpetual self-commentary with regard to himself, hence always missing out on his *true* position as commentator^28^. And the reason for this is clear: the effort to align the use of the first-person with the third-person has, as its ultimate consequence, the effect of installing a “division” of the subject, separating the one “who is speaking” from the one “who is spoken of.” Or more precisely: on such an account, when “I” speak about “myself,” the “I” and the “self” do not share the same reference (see also Silveira-Sales, 2007). And here, finally, we also encounter that “self-differing” movement (cf. infra) which, according to Lacan, fundamentally characterizes our position as speaking subjects: wanting to speak about myself in the first-person, and believing to express myself in the first-person present tense, I am irrevocably condemned to speak about myself in a third-personal way, that is, of referring to a “self” or “ego” from which, by the simple fact of this act of self-reference, I am immediately detached *qua* speaking subject. Putting this all together, Lacan asks:

Is the place that I occupy as subject of the signifier concentric or eccentric in relation the place I occupy as subject of the signified? That is the question. The point is not to know whether I speak of myself in a way that conforms to what I am, but rather to know whether, when I speak of myself, I am the same as the self of whom I speak (Lacan, 2006, p. 430).

Yet, despite the structuralist tropes invoked in dressing up this question, this is merely to offer a more convoluted version of the optical metaphor which sustains the detectivist idea of the unconscious: in the same way as the “eye,” in relation to its visual field, “sees everything except itself” (i.e. Lacan's “as a mode of opaqueness within the visible”); or, in the same way as the “I think,” in order to be able to accompany all my representations, cannot accompany its own act of representation, etc., the speaking subject can only refer to itself on the condition of remaining oblivious to its own position of enunciation^29^. The detectivist unconscious and the paradoxes generated by the idea of first-personal reference are then but two sides of the same coin: wherever self-knowledge is supposed in order to explain referential success, unconscious ignorance comes to haunt this very act of self-reference.

## Conclusion

The latter conclusion might be alarming for anyone who takes it upon himself to secure his existence by means of the Cartesian askesis, but for our present purposes, this also means that the account of the unconscious we get from Lacan's restatement of the cogito in terms of the enchanting formula “I am thinking where I am not, therefore I am where I am not thinking” (Lacan, 2006, p. 340) is, in the end, clearly parasitic on the detectivist picture of first-person authority. That is to say, it is indeed a correction *within* that picture, but not a correction *of* that picture. For it is not a matter of dispute that I can think and talk about myself and that the use of the first-person is the most ordinary way to do so. Whether I say I have the intention to go on a holiday or desire to finish this article, I indeed talk about myself, about my future intentions and current wishes. Nothing in particular has been decided by putting the issue in this way, because no philosophical account of this ability has been advanced either. However, does this necessarily mean, as Lacan seems to suggest, that in order to talk about myself, I have to *refer* to myself? Is speaking or thinking in the first-person to be considered as a referential activity in which I have to identify myself *with* myself and in which the “I” comes to function as a proper name and its psychological complements as descriptive predicates? Or, to put this in terms of Lacan's quarrel with the philosophies of consciousness: where in fact do we have to situate the “initial error of philosophy”? In the discussion about whether or not first-personal reference can ever attain that mythical moment of self-coincidence, or, indeed already, as Wittgenstein suggests, in the very idea of self-reference?

To clarify this issue, let us consider the following two sentences:

- It will rain
- I believe it will rain

The relevant question here is whether there is any difference with regard to the object of these two assertions. At a first impression, and because of the difference in their grammatical form, the two sentences at least *seem* to be different: the first one talking about the weather, the second one, on the other hand, containing a psychological proposition specifying my belief. However, according to Wittgenstein, by themselves, that is, without considering the context of use of these sentences, these grammatical appearances prove nothing. Yet, let us first develop Lacan's suggestion that sentences of the second form are referential descriptions which predicate something about an “ego” of which it is said that it “believes it will rain.” Here, it is strictly irrelevant whether this also means “alienating” oneself in language, the signifier, the mirror image or any other estranging medium held responsible for generating that unpalatable pathos so typical of Lacanian thinking; the same conclusion will hold for the Cartesian who does believe in the contemporaneity of “subject and consciousness.” Suppose, furthermore, that we have two people, the one affirming “I believe it will rain,” the other maintaining “No, I don't think so.” If we are to follow Lacan's account, these two speakers do not, and obviously even cannot, speak about the same thing: although, as we have seen, their true positions as speakers might remain unconscious, both can only refer to themselves, to the beliefs they report in describing them. But this is also to say, on the one hand, that the possibility of two speakers ever contradicting each other has been left behind, and indeed, as is now apparent, that the threat of solipsism has become unavoidable.

Assuming that even for the psychoanalyst dealing with psychic reality, the latter consequence is too high a price to pay, we have to reject the picture which seems to hold us captive here, which is in this case the assumption of semantical continuity across the first—and third-person uses of the psychological verb “believe.” That is, it is only because the sentence “I believe it will rain” is analyzed as a more private version of “He believes it will rain” that we seem to get into this philosophical mess of which, as we have tried to show, both unidentifiable subjects and communicative solipsism are the undesirable outcomes. Therefore, rejecting semantical continuity means recognizing that, grammatical appearances notwithstanding, the verb “believe” does not signify the same thing in the first- and third-person versions of “believing it will rain.” In the first-person present tense, “I believe” indeed *expresses* a belief, but this is not a belief referring to myself, nor to some meteorological belief-state, but quite evidently, a belief *about* the weather. But this also means, first of all, that, in contrast to the obvious difference between the sentences “He believes it will rain” and “It will rain,” sentences (i) and (ii) above are, from the perspective of their use and hence of their meaning, perfectly identical: the prefix “I believe” adds nothing to the meaning already found in “It will rain,” which is to say that it is entirely redundant. Yet, secondly, as per the Lacanian idea of the unconscious, this means that the subversive effort to lodge a moment of opacity within the temporal movement of self-reference is equally bound to failure: not because in saying “I believe it will rain” I am somehow magically protected from errors through misidentification, but simply because in this case there is no self-reference and hence no “identification” involved in the first place ^30^.

Yet, does this observation imply that the effort to offer a philosophical clarification of the adverbial meaning of unconscious should now be abandoned? It seems, on the contrary, that the only thing we have effectively abandoned is a distorted view on the first-person and, by implication, on the meaning of unconsciousness, which keeps producing nothing but conceptual incongruities. Recognizing what distinguishes speaking about oneself in the first person present indicative from third-personal reference is, first of all, to *reject* the detectivist picture of the unconscious in *all* its different guises, whether this means the deficiency of inner sense as in the earlier quote by Freud, or its more elaborate Lacanian version in terms of the paradoxes of self-reflection and first-personal reference. Speaking about someone else, I indeed refer to and identify someone by predicating something about his intentions and beliefs; but in my own case, none of these epistemic procedures are involved, and so the idea of the unconscious as involving beliefs, intentions or Cartesian subjects which would escape epistemic self-scrutiny cannot be involved either. However, dissociating the adverbial qualification of unconscious from failures of self-knowledge in this way, also means to bring out the truth of Freud's following incisive observation:

From what I have so far said a neurosis would seem to be the result of a kind of ignorance – a not knowing about mental events that one ought to know of. […] Now it would as a rule be very easy for a doctor experienced in analysis to guess what mental impulses had remained unconscious in a particular patient. So it ought not to be very difficult, either, for him to restore the patient by communicating his knowledge to him and so remedying his ignorance. […] If only that was how things happened! We came upon discoveries in this connection for which we were at first unprepared. Knowledge is not always the same as knowledge: there are different sorts of knowledge, which are far from equivalent psychologically. […] If the doctor transfers his knowledge to the patient as a piece of information, it has no result. […] The patient knows after this what he did not know before – the sense of his symptom; yet he knows it just as little as he did. Thus we learn that there is more than one kind of ignorance. We shall need to have a somewhat deeper understanding of psychology to show us in what these differences consist (Freud, 1966, pp. 280–281).

So, if, as well-attested by Freud, what it is for a desire or belief of mine to be unconscious is not primarily a matter of any straightforward first-personal epistemic shortcoming; moreover, if, on the contrary, knowledge as to my present unconscious inclinations seems perfectly compatible with the very existence of such inclinations, then what is the qualification “unconscious” a qualification *of* ? Or, as we put it earlier on in this paper, what exactly would be lost from our descriptive psychological repertoire if we opted to replace the term “unconsciously” by the more innocuous “unknowingly”? Would we effectively miss out on certain kinds of human behavior if our conceptual vocabulary was to be impoverished in this way?

We will conclude our article by arguing that this is indeed the case and that such examples can serve as an important step toward clarifying the adverbial meaning of unconsciousness. Here, both the subtle analyses of the phenomenon of “belief disavowal” by the French psychoanalyst Mannoni^31^ and Wittgenstein's grammatical remarks on the concept of unconscious^32^ can provide some decisive suggestions. To begin with the former: by means of the formula “I know quite well, but still …,” Mannoni aimed to capture a number of paradoxical, though seemingly entirely common situations in which someone appears to simultaneously entertain two contradictory beliefs. As, for example, the enlightened reader of horoscopes who “knows quite well” that cosmological predictions of future life events are sheer nonsense, yet still seems to take a strange pleasure in reading them anyway. In his own case, Mannoni observed that when the horoscope predicted that a certain day on which he had planned a move was indeed “particularly favorable for changes at home” (Mannoni, 1985, p. 20), he fell into strange fit of laughter. Yet, he also admits that his laugh would have been different if the horoscope had strongly advised against such domestic intentions. Or, in a similar example provided by Pfaller (2014, p.1), imagine that, while reading a newspaper, a friend arrives and says: “Excuse me, can I have a quick look at your newspaper? I know it's silly, but I just have to know the score from yesterday's game.” So, with regard to our present discussion, what needs to be highlighted in these examples is the paradoxical relation a subject seems to entertain with its beliefs (i.e., the importance of sports; the significance of horoscope predictions). In a way that is difficult to render conceptually transparent, these sorts of beliefs are never really believed in (“I know it's silly”; “I know these coincidences are meaningless”), yet in one way or another, they nonetheless exert a particular influence on their subjects (the compulsion to look at the paper—the pleasurable laughter). Moreover, as Pfaller argues (Pfaller, 2014), it is not so much their (trivial) content that is primarily of interest here, but rather the *form* in which people seem to refer to these beliefs. That is, as was already apparent from Mannoni's formula for this type of situations, its form is characterized by a rather unsettling coexistence of “better knowledge” and “belief.” The fidget sports fanatic knows quite well that yesterday's results are not important, but still he *has* to see them. Despite the better knowledge, and despite the ironical distance between him and his silly practice, he nevertheless acts *as if* sports are of utmost importance.

Importantly, in his own way, Wittgenstein seems to have seized on precisely these sorts of situations in order to justify a possible use of the adverb “unconsciously.” Starting from the observation that, as we have argued, in the first-person present indicative, the verb “believe” behaves in a different way compared to its third-personal form, Wittgenstein however also envisaged whether it would be possible to construct an intelligible use of “I believe” which would be symmetrical to the third-personal “He believes.” In order to create such a form, Wittgenstein proposes that we could modify the verb “believe” in the first person by adopting the expression “I seem to believe” or “going by my behavior, this is what I believe”:

My attitude to my own words is wholly different from that of others. I could find that variant conjugation of the verb, if only I could say “I seem to believe”. If I listened to the words issuing from my mouth, then I could say that someone else was speaking out of it. “Judging from my words, this is what I believe”. Now, it would be possible to think up circumstances in which this made sense (Wittgenstein, 2009, II§103-105).

And the sort of circumstances Wittgenstein deems relevant here are exactly those in which it would make reasonable sense to talk about “unconscious beliefs” or “unconscious intentions.” That is, “I seem to believe” is the expression I could use whenever, like in the situations described by Mannoni, I have to conclude, rather to my own surprise and despite better judgment, that I *seem* to behave as someone who believes this or that. Hence, what the qualification “unconscious” aims to capture in such instances is, on the one hand, the fact that we come to learn about these beliefs or desires in the same way as we would proceed in the case of others, that is, by observing and reporting on what is revealed to me in my own behavior and immediate reactions. Here, therefore, and in contrast to the use of the first person in the present indicative, it *does* make perfect sense to say that I “identify” myself as someone who unconsciously believes or desires certain things, for it is part of the very meaning of unconsciousness that I have to be informed about my beliefs and desires in such an alienated, third-personal way^33^. Yet, on the other hand, this also means, vindicating Freud's observation, that the idea of unconsciousness which is at stake here is in an important sense *non-epistemic*. On the contrary, since we have shifted to a symmetrical employment of psychological verbs, talk about my unconscious beliefs is precisely talk about what I, in principle, might come to *know* about myself through an attentive examination of my own expressive behavior or through the suggestions provided by the analyst. Nevertheless, as Freud had to acknowledge, acquiring such knowledge seems to be of little avail with regard to the efficacy of the beliefs in question. So, applied to Mannoni's idiom, what seems to single out those unconscious beliefs which would complement the expression “I know quite well, but still …” from those beliefs which, for example, are already expressed in the self-ascription “I know quite well,” cannot be related to a lack of detectivist introspection, nor to any paradox of self-reflection, but rather, as we suggested in our introduction, to something I seem unable to *do*. More precisely, although I do *express* these beliefs and desires in my actions, for example, in the urge to read the newspaper, or in the fading smile while reading disappointing cosmological prospects, what makes them specifically *unconscious* seems related to the fact that I am unable to express those beliefs and desires in a particular way, that is, as Finkelstein points out (Finkelstein, 1999, 2003), in expressive self-ascriptions while using the first person present^34^. Although I can describe my unconscious mental states through the use of symptomatic expressions like “I know, but still …”, I am unable to talk out about them in linguistic acts of self-expression, that is, by expressing my unconscious belief in simply *saying* “I believe.” In sum: on the expressivist view we have discussed here, an unconscious state of mind is not primarily a matter of something which I, qua epistemic subject, fail to *acknowledge*, but rather of a specific expressive position which I, as speaker, cannot *occupy*^35^.

## Author contributions

All authors listed have made a substantial, direct and intellectual contribution to the work, and approved it for publication.

### Conflict of interest statement

The authors declare that the research was conducted in the absence of any commercial or financial relationships that could be construed as a potential conflict of interest.

## Footnotes

^1^To be sure, it is not so much the historical philosopher Descartes that is at stake here, but a certain general picture of the human being that has emerged—for better or worse—from the Cartesian account, regardless of the question whether this picture accurately reflects Descartes's philosophy. Central to this picture are the distinction between extended bodies and minds, issues of “privileged” access to my own mind as opposed to others', the certainty of the first-person vs. the uncertainty of the third-person, a theory of ideas and related spectator-conception of consciousness, and so on. For some doubts about whether this “picture” accurately reflects Descartes' own theory, see Eshleman (2007).

^2^This idea regarding the *expressive* form of first-person avowals as different from the *descriptive* form of third-personal attributions is of course not only retrievable amongst ordinary language philosophers. For a similar treatment of this issue within the phenomenological tradition, see Husserl's section on “Expression and Meaning” in his *Logical Investigations* (Husserl, 1970) (see Renaudie, 2009, for a fine reading of this section). For a critical appraisal of the concept of “expression” in relation to the philosophical and psychoanalytic problematic of “representation,” see Deleuze (1990) and Deleuze and Guattari (1977).

^3^Most notably, David Finkelstein has presented some instructive clues as to how a philosophical reading of first-persona authority informed by the later Wittgenstein, can be of particular relevance for psychoanalytic conceptions of unconscious mentality. See, in particular, Finkelstein (1999). We will return to some of Finkelstein's suggestions later in our article.

^4^Paradigmatically for this case are Brentano's “four types of arguments” against the possibility of an “unconscious consciousness” in his *Psychology from an Empirical Standpoint* (Brentano, 2009, p. 81 *et passim*).

^5^For a detailed exposition of this notion and other related ones (e.g. “pre-reflective self-consciousness,” “first-personal-givenness,” “for-me-ness,” and so on), see Zahavi (2014).

^6^This idea of the necessary intervention of an active judgment in order to explain consciousness is even more explicit in Schelling's *System of Transcendental Idealism*, in particular in the section on “How the Self intuits itself as Sensing”: “The self *senses* when it finds in itself something opposed to it, namely, since the self is mere activity, a real negation of activity, or state of being affected. But to be that which senses, for itself, the (ideal) self must posit *in itself* that passivity which till now has been present only in the real; and this can undoubtedly occur only through *activity*” (Schelling, 1978, p. 61).

^7^The clearest formulation of the idea that the psychoanalytic concept of the unconscious is but the “immanent truth” of German idealism's concept of consciousness, can be found in Henry's *The Genealogy of Psychoanalysis*: “For the last word of the philosophy of consciousness, its limit and paradox, the zenith at which it turns against itself and self-destructs, is truly *the unconscious of pure consciousness itself*, the unconscious of “transcendental consciousness”. The historical moment of this turning occurs when German idealism, unable to found the principle on which it rests and eaten away from inside by this major inability, falls into nature philosophy, which is that idealism's truth, affirming nothing less than the unconscious of pure consciousness itself, namely, the unconscious of pro-duction” (Henry, 1993, p. 289). See also Redding (1999), Gardner (2012) and Feyaerts and Vanheule (2015).

^8^As noted by several commentators, Descartes uses the words “cogitation” and “cogitare” in both an extended as well as a more restricted sense compared to our ordinary understanding of those notions in the sense of “thinking” or “intellectual operations.” Extended because it also comprises, for example, the acts of imagination, sensing and willing. Restricted because Descartes retains only those acts which are immediately and absolutely “given” to consciousness. See Descombes (2004, pp. 176–189) and Anscombe (1981b).

^9^See Finkelstein (2003). Finkelstein refers to Wright's (2001) paper as the principal source of this term.

^10^Here I rely on Descombes' admirable analyses of what he calls “l'égologie cognitive” (Descombes, 2004, pp. 146–156).

^11^In his recent book *Self and Other* (2014), Zahavi argues, contrary to our suggestion, that a phenomenologically inspired account of consciousness and first-person authority does not amount to detectivism. The main argument being that from a phenomenological perspective, self-consciousness “is not brought about by some kind of reflection or introspection or higher-order monitoring,” but is in fact always already “an intrinsic feature of the primary experience” (p. 35). However, I am skeptical about this argument for the following reasons: first, “detectivism” is not primarily about what constitutes self-consciousness, that is, it doesn't single out a position with regard to the discussion between “one-level” or “two-level” accounts of self-consciousness. Rather, it is a philosophical account of what explains first-person authority, i.e., our seemingly effortless ability to say what we think, feel or intend to do. To put it in Sartrean terms, it is an account of *connaissance de soi*, rather than *conscience de soi*. Secondly, detectivism amounts to the claim that first-person authority is in some, very general sense, a matter of “finding out” what we think, hope or feel, regardless of the sort of *mechanism* that is invoked to describe this epistemic detection. In other words, if detectivism is to be rejected, then it is not only “introspection” which serves as an exemplary culprit, but *any* account that distinguishes two steps—one of knowing by detection, the other describing what one knows—in order to explain first-person authority. And this, I believe, is precisely the sort of account Zahavi advances when he writes about the transition between” pre-reflective self-consciousness” and “self-knowledge” in terms of “reflection” (p. 36).

^12^See Smith (1986), Thomasson (2000, 2006), Kriegel (2003), Shoemaker (1996) and Bar-On (2004).

^13^In short: if I know that I x whenever I x, it is because I x that I x whenever I x, and so on. As Leibniz put it: “(I)t is impossible that we should always reflect explicitly on all our thoughts; for if we did, the mind would reflect on each reflection, *ad infinitum*, without ever being able to move on to a new thought” Leibniz (1981, II, I, §19). As we have noted, this is one *locus classicus* to argue for the unconscious. This idea has been most rigorously conceptualized, with some modifications, by Lacan in his adoption of Bouasse's ‘optic mirror device'. See his “Remarks on Daniel Lagache's Presentation” in Ecrits (2006, pp. 543–574). For a clear exposition of this idea in relation to Kant's thesis on the impossibility of an ‘intellectual intuition' of the “I think,” see Zupancic (1996).

^14^Importantly, this is also why the defense of unconscious mentality is actually *more* rational and consistent within the framework of Cartesian higher-order theories than ideas about transparency or infallibility. From the moment one accepts two-act theories in order to explain first-person authority in a non-metaphysical way, that is, without implicitly assigning a different meaning to ordinary concepts like perception, belief or representation, one has to be prepared to endorse the idea that mental phenomena are indeed, as Freud put it, not necessarily what they appear to be (cf. supra). This would mean, for example, that I can “misperceive” my desire as a rational belief, can “misrepresent” my groundless anxiety as a reasonable intention, or that such mental phenomena can even go totally “unnoticed.”

^15^See Hacker (2005) for an extensive discussion. See also Bouveresse (1976, pp. 421–423).

^16^Most notably on the issue of whether or not expressive self-ascriptions can be truth-evaluable. See Rosenthal (1993) and Wright (1998) for typical formulations of this worry. See Alston (1967) and Finkelstein (2004, pp. 94–97) for responses.

^17^See Bar-On (2004, p. 200 *et passim*) for some further distinctions with regard to the ascriptive asymmetry.

^18^And since first-person ascriptions are treated alike by detectivists, i.e., as (second-order) reports or descriptions of (first-order) mental states indicated by their psychological terms, the only point of contention between, say, materialists and dualists, will be on the metaphysical status of these reported psychological entities [e.g., as (ir)reducible to brain-states] and the way such reports should be adjudicated [through, e.g., phenomenological or hetero-phenomenological methods]. For a representative sample of such discussions, see the collection of essays in *Phenomenology and the Cognitive Sciences* (2007, issue 1–2).

^19^This is why roughly the same line of argument can be found in Wittgenstein who, at the very least, does not share Sartre's phenomenological outlook: “Can we say that we have stripped bare the essential nature of the mind? “Formation of a concept.” Mightn't the whole thing have been treated differently?” (Wittgenstein, 1969, p. 45).

^20^To quote the famous lines from Hamlet: “Was't Hamlet wrong'd Laertes? Never Hamlet. / If Hamlet from himself be ta'en away, / And when he's not himself does wrong Laertes, / Then Hamlet does it not. / Who does it then? His madness.”

^21^One of Lacan's most clear expressions of this idea can be found in *Seminar 2: The Ego in Freud's Theory and in the Technique of Psychoanalysis*, p. 44: “The core of our being does not coincide with the ego. That is the point of the analytic experience, and it is around this that our experience is organized […]. But do you think that we should be content with that, and say—the *I* of the unconscious subject is not *me* [*moi*]? That is not good enough, because nothing […] implies the inverse. And normally you start thinking that this *I* is the real ego. You think that the ego is nothing but an incomplete, erroneous form of this *I*. In this way, you have accomplished the decentering essential to the Freudian discovery, but you have immediately reduced it.”

^22^To quote perhaps the most ardent defender of this view—Zizek: “Therein resides Lacan's achievement: the standard psychoanalytic theory conceives the Unconscious as a psychic substance of subjectivity (the notorious hidden part of the iceberg)—all the depth of desires, fantasies, traumas, and so on—while Lacan *de-substantializes* the *Unconscious* (for him, the Cartesian *cogito* is the Freudian subject), thereby bringing psychoanalysis to the level of modern subjectivity” (Zizek, 2016, p. 190).

^23^Cf. Freud (1940a, p. 159): “There is no need to characterize what we call “consciousness”: it is the same as the consciousness of philosophers and of everyday opinion.”

^24^Again, in Zizek's words: “[…] the unconscious is not some kind of pre-reflexive, pre-thetic, primitive substrate later elaborated upon by conscious reflexivity; quite the contrary, what is most radically “unconscious” in a subject is his self-consciousness itself, the way he reflexively relates to his conscious attitudes. The Freudian subject is thus identical to the Cartesian *cogito*, or, more precisely, to its later elaboration in Kantian-Hegelian self-consciousness” (Zizek, 2012, p. 554).

^25^See also Miller's diagnosis of this same “error”: “What is serious for Lacan is the logic of the signifier, that is to say the opposite of philosophy, inasmuch as every philosophy rests on the appropriateness, transparency, agreement, harmony of thought with itself. There is always some part hidden, in a philosophy, an I = I, which constitutes what Lacan called at some moment “the initial error in philosophy” which consists in privileging this equality and thus making one believe that the “I” is contemporary with itself, while its constitution is always after the emergence of its cause, of *petit a*” (Miller, 2006, pp. 22–23).

^26^See also Lacan's seminar on *Identification* where this idea is rendered even more explicit: “Therefore—as we have said—this sentence of: “I think” has the interest of showing us—it is the least we can deduce from it—the *voluntary dimension of judgment*” (Lacan, unpublished, p. 9, our italics). Elsewhere in the same seminar, Lacan coins the neologistic pun “mihilisme” to qualify this operation: “Thus, we see here, in short in our tongue, a sort of identification through the operation of a special significant tendency, that you will allow me to qualify as “mihilisme” in so far as to this act, this experience of the ego is referred” (Lacan, unpublished, p. 4).

^27^See also Lacan's “linguistic definition of *I* as signifier”: “In a concern for method, we can try to begin here with strictly linguistic definition of *I* as signifier, where it is nothing but the shifter or indicative that, qua grammatical subject of the statement, *designates* the subject insofar as he is currently speaking” (Lacan, 2006, p. 677, our italics).

^28^As indeed was already the conclusion drawn by that other eminent philosopher of reflexive paradoxes, i.e., Sartre: “Thus the consciousness that says ‘I think' is precisely not the consciousness which thinks. Or, rather, it is not its own thought which it posits by this thetic act” (Sartre, 2004b, p. 6). The only difference separating Lacan from Sartre with regard to this issue of first-personal reference is therefore related to a disagreement concerning the concept of “pre-reflexive consciousness”. Cf. supra.

^29^As Lacan puts it: “Is this not designed to make us question ourselves about what is at stake at this radical, archaic point that we must necessarily suppose to be at the origin of the unconscious, namely of this thing through which, in so far as the subject speaks, all he can do is to advance further along the chain, in the unfolding of enunciations, but that, directing himself toward the enunciations, by this very fact in the enunciating, he elides something which is properly *what he cannot know, namely the name of what he is qua enunciating subject*” (Lacan, unpublished, p. 62, our italics).

^30^Anscombe sums up this point as follows: “Getting hold of the wrong object *is* excluded, and that makes us think that getting hold of the right object is guaranteed. But the reason is that there is no getting hold of an object at all” (Anscombe, 1981a, p. 32).

^31^For Mannoni's theory of disavowal, see his two essays “Je sais bien, mais quand même …” and “L'illusion comique” in Mannoni (1985). For an English translation of the first essay, see Mannoni (2003, pp. 68–92). An important and lucid elaboration of Mannoni's account can be found in Pfaller (2014).

^32^For some of Wittgenstein's scattered remarks on a possible grammatical use of unconscious in relation to Moore's paradox, see Wittgenstein (1988, pp. 65–67). See also Descombes' brilliant discussion of this connection to which we are indebted for this point, in Descombes (2002).

^33^And not, contra Lacan, the other way around: i.e., because I always *have* to identify myself in one way or another, I necessarily *misidentify* myself qua unconscious subject.

^34^Other authors who have advanced similar views on the meaning of unconsciousness are Collins (1969), Rey (1988), and Moran (1997).

^35^Thanks to Dominiek Hoens, Russell Grigg and Vincent Descombes for their valuable suggestions
